# Obesity is associated with increased brain glucose uptake and activity but not neuroinflammation (TSPO availability) in monozygotic twin pairs discordant for BMI—Exercise training reverses increased brain activity

**DOI:** 10.1111/dom.70109

**Published:** 2025-09-10

**Authors:** Jaakko Hentilä, Jouni Tuisku, Ronja Ojala, Lihua Sun, Martin S. Lietzén, Heidi Virtanen, Riikka Lautamäki, Kalle Koskensalo, Lauri Nummenmaa, Eliisa Löyttyniemi, Semi Helin, Kirsi H. Pietiläinen, Jaakko Kaprio, Leo Lahti, Tarja Malm, Juha O. Rinne, Jarna C. Hannukainen

**Affiliations:** ^1^ Turku PET Centre, University of Turku Turku Finland; ^2^ Turku PET Centre, Turku University Hospital Turku Finland; ^3^ Huashan Institute of Medicine, Huashan Hospital, Fudan University Shanghai China; ^4^ Turku Collegium for Science, Medicine and Technology University of Turku Turku Finland; ^5^ Heart Centre, Turku University Hospital Turku Finland; ^6^ Department of Medical Physics Turku University Hospital Turku Finland; ^7^ Department of Psychology University of Turku Turku Finland; ^8^ Department of Biostatistics University of Turku and Turku University Hospital Turku Finland; ^9^ Radiopharmaceutical Chemistry Laboratory, Turku PET Centre University of Turku Turku Finland; ^10^ Obesity Research Unit, Research Program for Clinical and Molecular Metabolism, Faculty of Medicine University of Helsinki Helsinki Finland; ^11^ Abdominal Center, Obesity Center, Endocrinology University of Helsinki and Helsinki University Central Hospital Helsinki Finland; ^12^ Institute for Molecular Medicine Finland FIMM, HiLIFE, University of Helsinki Helsinki Finland; ^13^ Department of Computing University of Turku Turku Finland; ^14^ A.I. Virtanen Institute for Molecular Sciences University of Eastern Finland Kuopio Finland

**Keywords:** brain inflammation, cognitive function, insulin resistance, positron emission tomography, resting state functional magnetic resonance imaging

## Abstract

**Aims:**

Obesity is associated with increased insulin‐stimulated brain glucose uptake (BGU) which is opposite to decreased GU observed in peripheral tissues. Increased BGU was shown to be reversed by weight loss and exercise training, but the mechanisms remain unknown. We investigated whether neuroinflammation (TSPO availability) and brain activity drive the obesity‐associated increase in BGU and whether this increase is reversed by exercise training.

**Materials and Methods:**

Twelve monozygotic twin pairs mean age 40.4 (SD) years discordant for BMI (leaner mean 29.1 (SD) 6.3, heavier 36.7 (SD) 7.0 kg·m^−2^) performed 6‐month long exercise intervention. Insulin‐stimulated BGU during euglycaemic‐hyperinsulinaemic clamp, brain inflammation (translocator protein (TSPO) availability) and brain resting state activity were studied by [^18^F]FDG‐PET, [^11^C]PK11195‐PET, and fMRI, respectively. Cognitive function was assessed by an online survey.

**Results:**

Exercise training had no effect on insulin‐stimulated BGU, brain neuroinflammation (TSPO availability), or BMI. Exercise improved VO_2peak_, whole‐body insulin sensitivity, and cognitive function similarly in both groups (all, *p* <0.05) as well as decreased resting state brain activity in heavier co‐twins (*p* <0.05). At baseline, heavier co‐twins had worse whole‐body insulin sensitivity (*p* <0.01), increased BGU in the parietal cortex and caudatus, as well as increased resting state brain activity (both, *p* <0.05) and no difference in cognitive function. Leaner co‐twins had higher TSPO availability in white matter and the hippocampus (*p* <0.05).

**Conclusions:**

Exercise training had no effect on insulin‐stimulated BGU or neuroinflammation (TSPO availability) but it reversed increased resting state brain activity in heavier co‐twins. At baseline, obesity was associated with increased insulin‐stimulated BGU and resting state brain activity, independent of genetics.

## 1 INTRODUCTION

Obesity and physical inactivity are major risk factors for the development of insulin resistance (IR) and type 2 diabetes (T2D).^1^ IR in skeletal muscle, adipose tissue, and liver, followed by impaired pancreatic *β*‐cell function, plays a key role in the development of T2D.^2^ However, previous studies have highlighted the possible involvement of the brain in the development of IR/T2D.^3^, ^4^ Previous studies from our laboratory^5^, ^6^, ^7^ and elsewhere^8^ using [^18^F]FDG and positron emission tomography (PET) imaging have shown that brain glucose uptake (BGU) is increased upon insulin stimulation in people with obesity but not in normal weight healthy controls. This obesity‐associated increase in BGU has not been observed in the fasted state.^6^ It is unclear which brain cells increase their GU upon insulin stimulation. Rodent studies have shown insulin‐dependent glucose transporter 4 (GLUT4) expression in neurons, astrocytes, microglia, capillary endothelial, choroidal epithelial, and ependymal cells.^9^ In humans, insulin receptors have been found globally across the brain with higher density in the hypothalamus and cerebellum compared with the cerebellar cortex.^10^ Previous human studies using the FDG‐PET method have shown increased insulin‐stimulated BGU globally across the brain,^5^, ^6^, ^7^ which suggests that increased insulin‐stimulated BGU in people with obesity and IR is not limited to region‐specific cell types.

The proposed mechanisms for increased BGU include IR/T2D‐induced disruptions in the blood–brain barrier resulting in increased insulin leakage as well as neuroinflammation.^6^, ^11^ The rationale behind the neuroinflammation hypothesis is based on preclinical studies which suggest that a high‐fat diet induces astrogliosis, a neuroinflammatory response, especially in the hypothalamus.^12^ It has also been shown that astrocytes (inflammatory cells resident in the brain) contribute to the BGU.^13^ However, to our knowledge, no data exist on the effect of obesity on brain inflammation and its association with insulin‐stimulated BGU in humans. Nevertheless, some^14^, ^15^ but not all^16^ studies using MRI‐scan data suggest that obesity is associated with hypothalamic inflammation.

The functional significance of the increased insulin‐stimulated BGU associated with obesity/IR is currently unknown. Could it be possible that the brain is able to increase its GU to maintain whole‐body glucose homeostasis, thereby demonstrating an adaptive response? In previous FDG‐PET studies in people with obesity and IR, increased insulin‐stimulated BGU was found to be inversely associated with whole‐body insulin sensitivity,^7^, ^17^ suggesting that the BGU is oppositely regulated compared to peripheral tissues. We have previously shown that 2 weeks of high‐intensity interval training decreases insulin‐stimulated BGU, while simultaneously improving whole‐body and skeletal muscle insulin sensitivity in sedentary individuals with IR.^17^ Furthermore, previous studies have shown that bariatric surgery‐induced weight loss improves whole‐body insulin sensitivity and decreases insulin‐stimulated BGU in people with BMI >40 kg/m^2^.^5^, ^18^ Hence, previous studies suggest that increased BGU observed in obesity and IR may be reversible, but it is unclear whether this is driven by changes in neuroinflammation.

Functional MRI (fMRI) can reveal brain activity and temporal synchronisation between different brain regions and networks, and it is closely related to BGU.^19^, ^20^ The default mode network is one of the most studied resting state networks and is activated at rest, that is, in a task‐free environment.^21^ A previous study showed body adiposity‐associated increases in brain regions related to the default mode network.^22^ Furthermore, ineffective suppression of the default mode network was observed in individuals with obesity during an attention‐requiring task, which was associated with worse cognitive performance.^23^ Interestingly, 6 months of aerobic exercise training was shown to decrease the resting state activity of precuneus,^24^ which is an essential node of the default mode network.^25^ This suggests that exercise training may reverse the obesity‐associated increase in resting state brain activity in regions related to the default mode network.

The purpose of this study was to investigate whether the obesity‐associated increase in insulin‐stimulated BGU is linked to brain inflammation and altered resting‐state brain activity. To control for genetic and early environmental confounding, we studied monozygotic twin pairs discordant for BMI. We hypothesised that increased BGU is associated with neuroinflammation and altered brain activity, and that these changes would be reversed by regular exercise training.

## 2 MATERIALS AND METHODS

### 2.1 Ethics

This study is part of clinical exercise training intervention entitled ‘Systemic cross‐talk between brain, gut, and peripheral tissues in glucose homeostasis: effects of exercise training (CROSSYS, NCT03730610)’ performed at Turku PET Centre.

The study protocol, patient information and informed consent were approved by the Ethical committee of the Hospital district of Southern Western Finland (100/1801/2018/438§: approval date 23.11.2018). All the participants signed a written consent. The study was conducted according to the good clinical practice and the Declaration of Helsinki.

### 2.2 Study participants and study design

**FIGURE 1:**
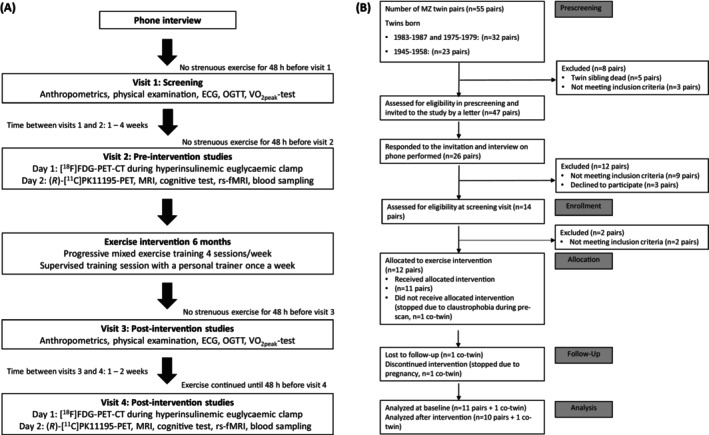
(A) Consort flow of the Crossys study. MZ = monozygotic. (B) Overview of the Crossys study protocol. CT, computed tomography; ECG, electrocardiography; MRI, magnetic resonance imaging; OGTT, oral glucose tolerance test; PET, positron emission tomography; rs‐fMRI, resting state functional magnetic resonance imaging; VO_2peak_, peak oxygen uptake, [^18^F]FD, 2‐deoxy‐2‐[^18^F]fluoro‐D‐glucose; (*R*)‐[^11^C]PK11195, ^11^C‐labelled R isomer of [1‐(2‐chlorophenyl)‐N‐methyl‐N‐(1‐methylpropyl)‐3‐isoquinolinecarboxamide].

The participants were monozygotic (MZ) twin pairs discordant for BMI (75% female, mean age 40.4 (SD) 4.5 years).^26^ Totally, 54 discordant twin pairs were identified from three unique population‐based longitudinal twin studies^26^, ^27^, ^28^, ^29^ and of these, 12 pairs were willing to participate, eligible, and enrolled in the study. Of these 12 pairs, 10 pairs finalised the exercise intervention period (Figure 1A). Of the leaner co‐twins, five had impaired fasting glucose (IFG) and two impaired glucose tolerance (IGT) and of the heavier co‐twins, seven had IFG and two IGT according to American Diabetes Association quidelines.^30^ Monozygosity of the twin pairs was determined as described.^31^ All participants were Finnish.

During the baseline measurements, anthropometric measurements, a physical examination, and an oral glucose tolerance test (OGTT) were performed after a 10 h fast, and physical performance tests were conducted (Figure 1B). Subsequently, functional and anatomical brain MRI scans and two PET imaging studies ((*R*)‐[^11^C]PK11195‐PET and [^18^F]FDG‐PET) were carried out on two separate days. After the baseline measurements, twin pairs exercised for 6 months, and the baseline measurements were repeated.^26^

### 2.3 Exercise performance tests, anthropometric measurements, and training intervention

Cardiorespiratory capacity (VO_2peak_) was measured by a stationary bicycle ergometer test (Ergoline 800 s, VIASYS Healthcare, Germany) and whole‐body fat percentage and lean mass by Inbody 720 (Biospace Co, Korea) at Paavo Nurmi Centre (Turku, Finland).^26^

Intervention consisted of two endurance, one resistance, and one high‐intensity interval exercise session per week for 6 months. Participants exercised at their place of residence and were supervised by a personal trainer once a week.^26^ The content of the training protocol is described in detail in Supplementary File File 1.

Participants were told to eat as they were used to and they filled food diaries from three consecutive days before, in the middle, and at the end of the intervention.

### 2.4 Euglycaemic‐hyperinsulinaemic clamp, [ ^18^F]FDG–PET/CT scan, and T1 weighted MRI scan

BGU was studied during the euglycaemic‐hyperinsulinaemic clamp with [^18^F]FDG by PET/CT (Discovery MI (DMI), GE Healthcare, USA).^26^ Whole body insulin sensitivity (M‐value) was calculated as previously described.^32^, ^33^ After the steady state was achieved in the euglycaemic‐hyperinsulinaemic clamp, 150 MBq of [^18^F]FDG was injected into the antecubital vein via a catheter, and brain scanning was started for 40 min. Plasma radioactivity for the input function was measured from arterialised blood samples.

To achieve anatomical reference images for PET and rs‐fMRI analysis, T1 weighted brain MRI scan was performed.^26^ Visceral fat mass was analysed as previously described.^26^, ^33^

### 2.5 [ ^11^C]PK11195‐PET/CT scan

TSPO availability to assess brain‐specific inflammation was measured with [^11^C]PK11195 (350 MBq) by 60 min PET scan without insulin stimulation on a different day than FDG scan.^26^

### 2.6 PET‐image and rs‐fMRI analysis and modelling

PET‐image and rs‐fMRI analysis as well as modelling are described in Supplementary File File 2.

### 2.7 Cognitive function test

Cognitive function was assessed by an online survey using Gorilla Experiment Builder (gorilla.sc) on a standard desktop computer^26^ and the test is described in detail in Supplementary File File 3.

### 2.8 Statistical analysis

Statistical tests were performed by the SAS System (version 9.4 for Windows SAS Institute, Cary, NC, USA) as two‐sided, and *p*‐values less than 0.05 were considered statistically significant. The normal distribution of the data was evaluated visually from Q–Q plots and histograms as well as studentized residuals from the model. Statistical analyses were conducted using a linear mixed model for repeated time points using compound symmetry covariance structure. The model included twin as a statistical unit, time (PRE and POST intervention) and twin group (leaner and heavier co‐twin) as within‐factors and their interaction term (time×group).

Used estimation was restricted maximum likelihood, which also allows participants with missing data to be included. If there was a significant time × group effect, the same model was used to determine the within twin‐group effects over time. The baseline difference between the co‐twins was estimated from the same model using pre‐intervention data.

#### 2.8.1 Power calculations

It was not possible to calculate the sample size for the main outcome variables BGU and TSPO availability due to non‐existing twin data. Originally, the sample size calculations for this study were based on liver fat content, M‐value, and VO_2peak_ results from the earlier cross‐sectional studies in twins discordant for physical activity and fitness as previously described in detail.^26^ According to the liver fat calculations, 22 pairs were aimed to recruit, but for variables with lower deviation, such as M‐value and VO_2peak_ even under 10 pairs would have been sufficient.

## 3 RESULTS

### 3.1 Anthropometrics, physical fitness, glucose and lipid profile

At baseline, heavier co‐twins had significantly higher body adiposity (difference 33%, *p* <0.001) as well as lower cardiorespiratory fitness (VO_2peak_) (difference 27%, *p* = 0.003) and whole‐body insulin sensitivity (M‐value) (difference 63%, *p* = 0.007) compared with their leaner co‐twins (Table 1). Furthermore, heavier co‐twins had a worse blood glucose homeostasis profile compared with their leaner co‐twins (Table 1).

**TABLE 1:** Participant characteristics before (Pre) and after (Post) the exercise intervention. Data are expressed as model‐based means [95% CIs].

	Leaner co‐twins		Heavier co‐twins		*p*‐value		
	Pre (*n* = 12)	Post (*n* = 10)	Pre (*n* = 12)	Post (*n* = 11)	Baseline	Time	Time × group
Sex	8 female/4 male pairs						
Anthropometrics							
Age	40.4 [37.5;43.4]		40.4 [37.5;43.4]				
Weight (kg)	86.4 [72.4;100.4]	86.9 [72.6;101.2]	108.7 [94.2;123.3]	108.0 [93.1;122.9]	**0.001**	0.948	0.374
BMI (kg/m^2^)	29.1 [25.2;33.0]	29.3 [25.3;33.2]	36.7 [32.7;40.7]	36.4 [32.4;40.4]	**0.0006**	0.921	0.407
Whole‐body fat (%)	30.4 [21.3;39.6]	29.5 [20.3;38.7]	40.6 [36.5;44.7]	40.0 [35.9;44.1]	**0.0005**	0.370	0.718
Lean mass (kg)	33.1 [30.0;36.3]	33.9 [30.6;37.2]	35.9 [31.9;39.8]	36.2 [32.1;40.3]	**0.003**	0.140	0.102
Visceral fat mass (kg)	3.38 [2.13;4.64]^a^	3.22 [2.03;4.42]	5.83 [4.74;6.93]^c^	5.46 [4.42;6.50]^b^	**0.002**	0.067	0.280
Systolic BP (mmHg)	131.4 [120.4;143.4]	122.3 [114.1;131.1]	136.1 [128.4;144.4]	126.8 [121.2;132.7]	0.375	**0.011**	0.983
Diastolic BP (mmHg)	80.1 [73.3;86.9]	77.1 [71.2;83.0]	86.9 [80.2;93.5]	78.3 [72.5;84.0]	0.074	**0.017**	0.091
VO_2peak_ (mL/kg/min)	32.4 [26.9;37.8]	35.1 [29.9;40.2]	25.6 [23.2;28.0]	28.3 [26.1;30.6]	**0.003**	**0.001**	0.935
hs‐CRP (mg/L)	0.81 [0.41;1.61]^b^	0.56 [0.21;1.47]^c^	1.42 [0.75;2.70]^b^	1.14 [0.46;2.85]^d^	**0.005**	0.296	0.450
Glucose profile							
Fasting glucose (mmol/L)	5.5 [5.2;5.7]	5.5 [5.2;5.7]	5.7 [5.4;5.9]	5.8 [5.6;6.1]	0.388	0.367	0.418
Fasting insulin (mU/L)	6.6 [5.1;8.7]	6.3 [4.3;9.3]	11.1 [8.7;14.2]	9.9 [6.9;14.1]	**0.006**	0.502	0.711
HOMA‐index	1.60 [1.19; 2.16]	1.53 [1.01; 2.31]	2.78 [2.15; 3.58]	2.55 [1.79; 3.63]	**0.013**	0.589	0.866
HbA1c (mmol/mol)	34.9 [32.9;36.9]	34.7 [32.2;37.1]	36.5 [35.0;38.0]	36.0 [34.2;37.7]	**0.047**	0.581	0.675
M‐value (μmol/kg/min)	37.5 [28.0;47.0]^a^	46.9 [31.7;62.1]^b^	23.0 [16.1;29.9]^a^	31.4 [20.4;42.3]^c^	**0.007**	**0.022**	0.82
Lipid profile							
Triglycerides (mmol/L)	0.79 [0.60;1.02]	0.77 [0.64;0.94]	1.20 [0.81;1.78]	1.08 [0.81;1.43]	**0.040**	0.536	0.491
FFA (mmol/L)	0.52 [0.36;0.70]^a^	0.49 [0.34;0.66]	0.59 [0.43;0.78]^a^	0.56 [0.42;0.72]	0.328	0.666	0.956
Total cholesterol (mmol/L)	4.37 [3.70;5.15]	4.45 [3.88;5.11]	4.68 [4.01;5.46]	4.57 [4.02;5.20]	0.197	0.972	0.148
LDL (mmol/L)	2.77 [2.28;3.36]	2.77 [2.30;3.32]	3.12 [2.59;3.77]	3.03 [2.54;3.62]	0.101	0.721	0.525
HDL (mmol/L)	1.40 [1.21;1.62]	1.49 [1.32;1.69]	1.22 [1.05;1.42]	1.24 [1.09;1.41]	0.086	0.133	0.109

*Note*: *P*‐value for baseline describes the difference between heavier and leaner co‐twins before exercise intervention. *P*‐value for time describes the change from PRE to POST in all participants. *P*‐value for time × group interaction describes the change difference between heavier and leaner co‐twins from pre to post.

Abbreviations: BP, blood pressure; FFA, free fatty acid; Hba1c, glycated haemoglobin; hs‐CRP, high‐sensitivity C‐reactive protein; HDL, high density lipoprotein; LDL, low density lipoprotein; M‐value, whole‐body insulin sensitivity; VO_2peak_, cardiorespiratory fitness; Systolic BP, hs‐CRP, fasting insulin, HOMA‐index, triglycerides, total cholesterol, LDL, and HDL were log‐transformed for the time and time*group analysis whereas FFA was square root‐transformed for the time and time*group analysis. Visceral fat mass, hs‐CRP, HOMA‐index, Hba1c, triglycerides, total cholesterol, LDL, and HDL were log‐transformed whereas FFA and lean mass were square root‐transformed for the baseline comparison; hs‐CRP was measured before [^11^C]PK11195‐scan in a post‐prandial state.

^a^ *n* = 11.

^*b*^ *n* = 10.

^c^ *n* = 9.

^d^ *n* = 8.

Blood insulin values during the clamp were slightly higher post intervention at 120 min after the start of the clamp (*p* = 0.022) (Supplementary File File 4).

Exercise intervention improved cardiorespiratory fitness (9.4%, *p* = 0.001) and whole‐body insulin sensitivity (29.4%, *p* = 0.022) as well as lowered systolic (9.7 mmHg, *p* = 0.011) and diastolic (5.8 mmHg, *p* = 0.017) blood pressure similarly in leaner and heavier co‐twins (Table 1). Training decreased visceral fat mass in heavier co‐twins (0.37 kg, *p* = 0.029; Table 1) but the decrease was not statistically significant compared with the change in leaner co‐twins (time × group *p* = 0.280). Training had no effect on whole‐body fat percentage (*p* = 0.370) in either group. There was no statistically significant difference between the co‐twins in the total energy intake at PRE or POST intervention (data not shown).

### 3.2 Insulin‐stimulated brain glucose uptake

**FIGURE 2:**
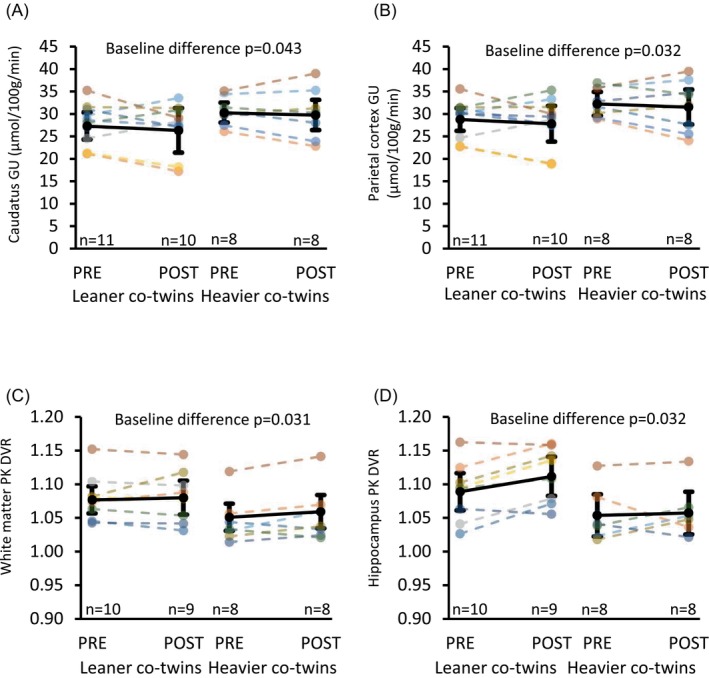
Insulin‐stimulated glucose uptake (GU) in (A) caudatus and (B) parietal cortex measured by [^18^F]FDG‐PET/CT during euglycaemic hyperinsulinamic clamp and brain translocator protein (TSPO) availability measured by [^11^C]PK11195 distribution volume ratio (DVR) before (PRE) and after (POST) the exercise intervention in heavier and leaner co‐twins in C) white matter and D) hippocampus. In (A) and (B) as well as in (C) and (D) figures, twin pairs share the same colour in dashed lines and solid black line depicts model‐based mean with 95% confidence intervals. *p*‐values indicate statistical significance between leaner and heavier co‐twins at baseline.

At baseline, heavier co‐twins had higher BGU globally in each ROI set compared with leaner co‐twins, but the difference reached statistical significance only in parietal cortex (difference 11%, *p* = 0.032) and caudatus (difference 9%, *p* = 0.043; Figure 2A,B; Supplementary File File 5). Exercise training had no effect on BGU.

### 3.3 Brain inflammation measured by TSPO availability

At baseline, leaner co‐twins had higher TSPO availability at white matter (2.2% difference, *p* = 0.031) and hippocampus (3.2% difference, *p* = 0.032) compared with heavier co‐twins (Figure 2C,D; Supplementary File File 5). Interestingly, TSPO availability in the hippocampus, white matter, and whole brain correlated positively with whole‐body insulin sensitivity and VO_2peak_ as well as negatively with CRP, BMI, and visceral fat mass (Supplementary File File 6).

### 3.4 Resting state‐functional MRI

**FIGURE 3:**
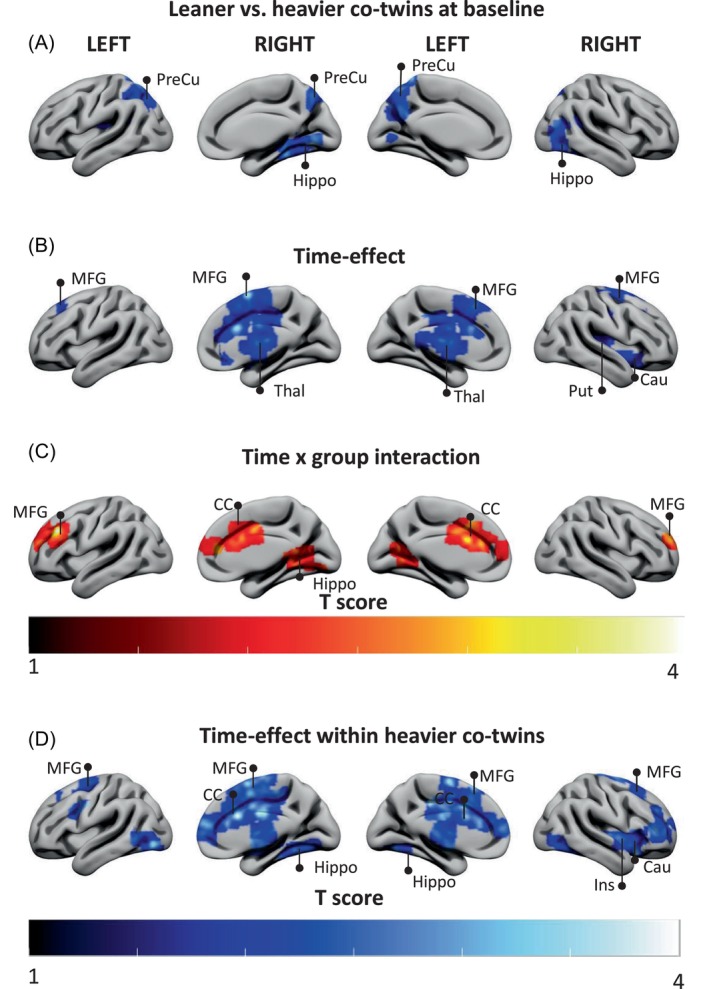
Resting state brain activity (BOLD signal) measured by functional MRI at resting state. In (A) blue voxels depict brain areas where heavier co‐twins had higher brain activity compared with their leaner co‐twins at baseline, (B) blue voxels depict areas where brain activity was decreased post training in the whole sample, (C) red and yellow voxels depict brain areas where training response was greater in heavier co‐twins compared with their leaner co‐twins, and (D) blue voxels depict areas where brain activity was decreased post training within heavier co‐twins. All data are FDR‐corrected at *p* <0.05. CC, Cingulate Cortex; Cau, Caudate; Ins, Insula; Hippo, Hippocampus; MFG, middle frontal gyrus; Put, Putamen; PreCu, Precuneus; Thal, Thalamus.

At baseline, heavier co‐twins demonstrated higher resting state brain activity (BOLD signal) in the precuneus, which is a key node of the brain default mode network compared to the leaner co‐twins (*p* <0.05; Figure 3A). Training decreased resting state brain activity in the medial frontal gyrus, thalamus, caudate, and putamen (Figure 3B) and, interestingly, the training response was different between the groups (*p* <0.05; Figure 3C) showing a statistically significant decrease only in heavier co‐twins (*p* <0.05; Figure 3D).

### 3.5 Cognitive function

**FIGURE 4:**
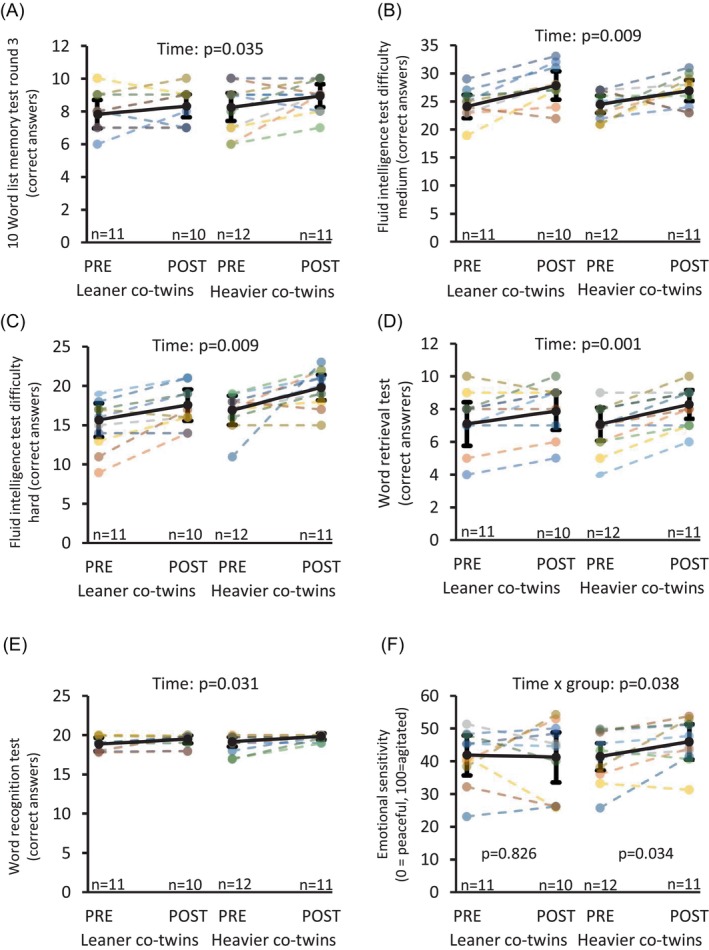
Cognitive test results that showed statistically significant improvement post training. (A) CERAD 10 Word list memory task type test from the third round: (B) and (C) fluid intelligence tests: (B) degree of difficulty medium (C) degree of difficulty hard. (D) Word retrieval test, (E) Word recognition test, (F) Emotional sensitivity, pleasant pictures shown (reaction: 0 = peaceful – 100 = agitated). Twin pairs share the same colour in dashed lines and solid black line depicts model based mean with 95% confidence intervals. *P*‐values for time indicate statistical significance for the change from pre to post in the whole sample. *P*‐value for time x group interaction indicates statistical significance for the change difference between heavier and leaner co‐twins from pre to post.

At baseline, there was no difference in cognitive performance between the leaner and heavier co‐twins (Supplementary File File 3). Training improved performance in memory encoding and retrieval on average by 8% (*p* <0.05, Figure 4A–C) as well as in fluid intelligence tests on average by 14% (*p* <0.05, Figure 4D,E) similarly in leaner and heavier co‐twins. When participants were shown pleasant pictures, the intervention shifted the emotional reaction towards agitation more in heavier co‐twins (*p* = 0.034) compared with their leaner co‐twins (time × group: *p* = 0.038, Figure 4F).

## 4 DISCUSSION

This study on MZ pairs discordant for body weight showed that independent of genetics, higher BMI is associated with higher insulin‐stimulated BGU. Contrary to our initial hypothesis, the increased insulin‐stimulated BGU in caudatus and parietal cortex was not reversed by exercise training. Additionally, BGU did not associate with TSPO availability, which was measured to assess brain inflammation. Vice versa, we observed that TSPO availability in white matter and hippocampus was increased in leaner co‐twins compared with their heavier co‐twins. Interestingly, we observed higher resting brain activity in heavier co‐twins compared with their leaner co‐twins, and this increase in brain activity was reversed by exercise training intervention. Training intervention also improved memory encoding, fluid intelligence, cardiorespiratory fitness, and whole‐body insulin sensitivity but had no effect on body weight similarly in both co‐twin groups.

Previous studies have shown that BGU is increased upon insulin stimulation in people with obesity and IR but not in normal weight people.^5^, ^6^ Furthermore, increased insulin‐stimulated BGU has been associated with worse whole‐body insulin sensitivity^7^, ^34^ but the underlying mechanisms are unknown. The increase in insulin‐stimulated BGU has been global across the whole brain^5^, ^6^ but there have been regional differences in absolute BGU levels. For example, in the study by Tuulari et al., the BGU increase upon insulin stimulation was highest in the right caudate nucleus.^5^ In this study, we showed that independent of genetics, increased BMI resulted in higher insulin‐stimulated BGU in all analysed regions, but the difference was statistically significant only in the caudate nucleus and parietal cortex. This aligns to some extent with the results by Tuulari et al. and might suggest the caudate nucleus being most sensitive to obesity‐associated changes. The reason for not reaching statistical significance in all brain regions in this study may be due to the small number of study participants and studies with larger sample sizes are warranted.

Even though we saw obesity‐associated region‐specific increases in insulin‐stimulated BGU, the exercise intervention did not decrease BGU in these regions in neither of the co‐twin groups. We have previously shown that only 2 weeks of high‐intensity interval training decreases insulin‐stimulated BGU in middle‐aged people with insulin resistance.^17^ Furthermore, decreased BGU in people with BMI >40 kg/m^2^ after bariatric surgery was observed.^5^ In these previous studies, the intervention induced weight loss, which was not observed in this study, which could explain the discrepancy between the studies. In the current study, the heavier twins were obese but metabolically quite healthy and healthier compared to the participants in the previous studies showing decreased BGU after weight loss.^5^, ^17^ Furthermore, no weight loss was observed in the current study. Also, higher circulating insulin levels were observed during the hyperinsulinemic euglycemic BGU measurement (at time point 120 min) in both groups post‐training compared to baseline. The increase in insulin levels may be a response to improved glucose sensitivity in the muscles, whereas type 2 diabetic individuals may compensate for improved whole‐body insulin sensitivity by maintaining or decreasing insulin secretion, which was already increased at baseline.^35^ Thus, in the current study, the circulating insulin stimulus to the brain was similar or partly increased during the BGU measurement post training, which may explain why not as clear intervention‐induced changes were observed as in previous studies.

Preclinical^36^ and some^14^, ^15^ but not all^16^ clinical studies have suggested that a high‐fat diet and obesity induce inflammation in the brain (especially in hypothalamus). We hypothesised that the increased insulin‐stimulated BGU observed in previous studies^5^, ^6^, ^7^, ^8^ could be an obesity‐induced neuroinflammatory response. In this study, we measured neuroinflammation (TSPO availability) with PET imaging using radio tracer [^11^C]PK11195.^37^ This radioligand binds to the TSPO protein that has been mainly found in microglia and astrocytes in the human brain. In the normal brain, TSPO expression is low. However, upon pro‐inflammatory stimuli, the expression of TSPO increases in microglia and astrocytes.^38^, ^39^ Thus, hypothetically, as brain‐resident astrocytes and microglia respond to obesity‐induced pro‐inflammatory stimuli, their energy expenditure and abundance would increase, which would be seen as an increased BGU. This hypothesis is supported by a previous preclinical [^18^F]FDG‐PET study showing that astrocytes also contribute to the BGU in addition to the BGU by neurons.^13^ Also, preclinical studies suggest that high‐fat diet‐induced obesity increases the amount of astrocytes in the mouse brain.^12^

However, contrary to our hypothesis, we showed that leaner co‐twins had higher TSPO availability in white matter and hippocampus. Furthermore, we found that whole brain TSPO availability correlated negatively with BMI, CRP, and visceral fat mass and positively with whole‐body insulin sensitivity and VO_2peak_. Thus, higher TSPO availability was associated with biomarkers of better metabolic health. This result aligns with a previous study where Tuisku et al. combined brain scans, measured with second generation TSPO radioligand [^11^C]PBR, from three different PET centres (*n* = 140) and observed a significant negative correlation between BMI and TSPO availability.^40^ A recent study showed that during neuroinflammation, TSPO expression is increased in rats and mice but not in humans.^41^ That study emphasised that TSPO signal in human brain should be interpreted as an indicator of glial cell density rather than glial cell activation. Applying this idea to our findings would suggest that obesity reduces the number of glial cells in the brain.

TSPO has high binding‐affinity to cholesterol.^42^ In the current study, there was a tendency for higher LDL and total cholesterol levels in heavier co‐twins, which might result in fewer TSPO binding sites and thus TSPO availability in heavier co‐twins.

Because TSPO is a mitochondrial protein with many functions, it may be speculated that obesity decreases mitochondrial content or functional properties also in the brain.^43^ The derangement of mitochondrial function is a common feature observed in peripheral tissues in people with obesity and contributes to whole‐body insulin resistance.^44^

Interestingly, we showed that heavier co‐twins had higher brain activity at resting state at baseline in default mode network specific brain regions (precuneus). This aligns with a previous study showing that brain activity in regions related to the default mode network is more active in people with obesity compared to age‐ and sex‐matched normal‐weight controls.^22^ In the current study, insulin‐stimulated BGU also tended to be higher in the precuneus in heavier co‐twins (data not shown). This suggests that the higher BGU in heavier co‐twins may reflect increased energy consumption due to higher neural activity. This is supported by a previous study which showed a positive correlation between BGU and resting state brain activity.^20^ We also showed that the intervention decreased resting state brain activity more in heavier compared with leaner co‐twins. This finding is consistent with a previous study showing that 6 months of exercise training decreased precuneus activity in overweight participants.^24^

Obesity is associated with worse cognitive function, especially executive function and working memory.^45^ Cognitive performance did not differ between the groups at baseline in the current study. However, training improved memory encoding and retrieval similarly in both groups. In addition, fluid intelligence was improved by the intervention. One possible mechanism by which exercise training improves cognitive performance may be mediated by exerkine brain derived neural factor (BDNF) that increases post exercise.^46^ In the brain, BDNF induces neurogenesis which is beneficial for the brain function and cognitive performance.^47^ Another possible explanation for the improved cognitive function after exercise training may be improved ability to switch from resting state network (e.g., from default mode network) to task related/attention requiring brain network.^23^ To support this postulation, we showed that exercise training decreased brain activity at resting state in regions that are part of default mode network (medial prefrontal cortex, precuneus and insula) and this effect was larger in heavier co‐twins. However, we should have measured brain activity by fMRI while changing the test environment from rest to cognitive tasks to confirm this. The benefits of exercise training on cognitive function are widely shown.^48^ This study further suggests that exercise training, even without weight loss, can benefit cognitive function, and this benefit is not limited to older populations with cognitive impairments. The education level between the co‐twins was not significantly different and thus is not a confounding factor in this study (data not shown). However, we cannot rule out the learning effect (PRE vs. POST testing) in this study, because a control group was not included.

The strength of this study is that we were able to study the effect of increased body weight while controlling for genetic factors by studying MZ‐twin pairs discordant for BMI. Furthermore, the exercise intervention was well planned, which manifested as high training adherence (approximately 80%) as well as improved cardiorespiratory fitness and whole‐body insulin sensitivity. In addition, the changes induced by the intervention are not confounded by body weight loss. We also used state‐of‐the‐art methods to measure non‐invasively insulin‐stimulated BGU. We agree that some of the results may have been confounded by nutrition, which was not controlled during the intervention. The analysis of the three‐day food diaries revealed no significant differences in energy and macronutrient intake between the co‐twins at baseline or post training, nor did we find a decrease in BMI. As BMI did not decrease, the training‐induced energy deficit was probably reversed by a simultaneous increase in energy intake. It should be noted that people tend to underreport their energy intake, and this is proportional to a higher BMI,^49^, ^50^ which may explain these findings.

The limitation of this study is that, even though there was a substantial mean level BMI difference (7.6 kg/m^2^) between leaner and heavier co‐twins, there was heterogeneity between the twin pairs which may cause confounding variability in the data. More precisely, in some twin pairs both individuals were with obesity, and in one twin pair both were only overweight and close to normal weight. In addition, on average, the leaner co‐twins were overweight. Ideally, the intra‐pair difference in each pair would have been as high as possible, and the BMI of the leaner co‐twins would have been <25 kg/m^2^ and that of heavier co‐twins >25 kg/m^2^. Furthermore, we had eight pre‐menopausal female twin pairs and four male pairs in our study, which may induce some heterogeneity for the exercise response (time‐effect) because pre‐menopausal women are more insulin sensitive compared to men.^51^ However, the comparisons at baseline and for the exercise response between groups (time × group) were made between co‐twins and thus this excludes the effect of sex in these comparisons.

We could not calculate the sample size for the main outcome variables (BGU and TSPO availability) due to non‐existing twin data; the total number of twin pairs in this study was smaller than we aimed to recruit, and some measurements were not successful, which yielded a modest sample size. Furthermore, in the light of new evidence^41^ which we were not aware of at the time of clinical study conduct, [^11^C]PK11195 is not the most optimal radiotracer to measure neuroinflammation in the human brain. Thus, this study should be replicated with a larger sample size and a more suitable radiotracer.^41^

In this unique twin study, obesity was associated with increased insulin‐stimulated glucose uptake in the caudate and parietal cortex and with elevated resting‐state brain activity in regions of the default mode network. Regular exercise training reversed the obesity‐associated increase in brain activity, but had no effect on insulin‐stimulated BGU or neuroinflammation (TSPO availability). Furthermore, no association was observed between BGU and TSPO availability, suggesting that increased glucose uptake is not directly driven by neuroinflammation.

## AUTHOR CONTRIBUTIONS

Planning of the study design (JCH, TM, LL and JOR). Participant recruitment and data collection (JH, RO, MSL). PET data analysis (JT and JH). Resting state fMRI data analysis (LS and JH). Planning the cognitive function test (LN). Planning of the statistical analyses (EL). Responsible physician (RL). MRI scans (KK). Visceral fat mass analysis (HV). Radiotracer production [^11^C]PK11195 (SH). Responsible for the twin cohort (KHP and JK). JH wrote the first manuscript, which was then revised by JCH. The revised version of the manuscript was read, commented on, and approved by all authors.

## FUNDING INFORMATION

This study is financially supported by: Academy of Finland (JCH decision 317 332), Hospital District of Southwest Finland, the Finnish cultural foundation (JCH, JH and MSL), Kyllikki and Uolevi Lehikoinen foundation (JH), the Diabetes research foundation of Finland (JCH, JH, RO and MSL), Varsinais‐Suomi Regional Fund (JCH, RO and JH), Maud Kuistila memorial foundation (JH), Maija and Matti Vaskio Foundation (MSL), Emil Aaltonen Foundation (MSL), the Turku Finnish University Society (RO), Turku University Foundation (RO) and University of Turku Doctoral Programmes in Clinical Research (RO). JK has been supported by the Academy of Finland Center of Excellence in Complex Disease Genetics (grant # 352792 to Jaakko Kaprio).

## CONFLICT OF INTEREST STATEMENT

The authors declare no conflict of interest.

## PEER REVIEW

The peer review history for this article is available at https://www.webofscience.com/api/gateway/wos/peer-review/10.1111/dom.70109.

## Supporting information


**Supplementary File 1:** Progressive training intervention for 26 weeks.

Supplementary File 2. PET‐image analysis and modelling

**Supplementary File 3.** Cognitive function test results before (PRE) and after (POST) the exercise intervention.

**Supplementary File 4.** Blood glucose and insulin levels measured during euglycaemic hyperinsulinemic clamp before (PRE) and after (POST) the exercise intervention.

**Supplementary File 5.** Insulin‐stimulated glucose uptake and TSPO availability in different regions of interest (ROI) of brain before (PRE) and after (POST) the intervention.

**Supplementary File 6.** Statistically significant Pearson correlation coefficients between translocator protein (TSPO) availability measured by PK distribution volume ratio (PK DVR) and main outcome measures at baseline (Pre).

## Data Availability

The dataset generated and analysed during the current study are not publicly available in order to protect the individual privacy. However, the data is available from the corresponding author on a reasonable request for researchers who have institutional review board/ethics approval and an institutionally approved study plan.
