# Supplementary material for: Obesity is associated with increased brain glucose uptake and activity but not neuroinflammation (TSPO availability) in monozygotic twin pairs discordant for BMI—Exercise training reverses increased brain activity
Source: Diabetes Obes Metab. 2025 Sep 10;27(12):7097–109. doi: 10.1111/dom.70109 (PMC12587225; doi:10.1111/dom.70109)
Supplement: Supplementary file 1 — Progressive training intervention for 26 weeks. [file DOM-27-7097-s006.docx]

**Supplementary file 1:** Progressive training intervention for 26 weeks.

| **ENDURANCE TRAINING (twice a week: one Session 1 and one Session 2)** | | | | | | | | | | | |
| --- | --- | --- | --- | --- | --- | --- | --- | --- | --- | --- | --- |
|  | *Weeks 1-2* | | *Weeks 3-4* | *Weeks 5-6* | | *Weeks 7-9* | | *Weeks 10-19* | | *Weeks 20-26* | |
| Session 1 | 70% HR_max_ 30 min | | 70% HR_max_ 35 min | 70% HR_max_ 40 min | | 70% HR_max_ 45 min | | 75% HR_max_ 45 min | | 80% HR_max_ 45 min | |
| Session 2 | 60% HR_max_ 40 min | | 60% HR_max_ 50 min | 60% HR_max_ 60 min | | | | | | | |
| **HIGH INTENSITY INTERVAL TRAINING (once a week one type of training)** | | | | | | | | | | |  |
| *Type* | *Completion method* | | | | | | *Content of the training* | | | |  |
| **Circuit** | Perform 4 rounds of the movements in chronological order. Every round, spend 1 minute in each movement: do as many repetitions as you can in 40 seconds and then rest for 20 seconds. Rest 1 minute between the rounds after completing all movements (1-6).  **Above 80% of HR_max_** | | | | | | 1. Lunges 2. X-jump/jumping rope/mountain climber 3. Back extension 4. Abdominal crunches (obiqual) 5. Burpee 6. Hip thrust 7. Rest | | | |  |
| **Cross training** | First, perform 12 minutes of section A (switch between two movements). Rest 1 minute.  Second, perform 6 minutes of section B. Rest 1 minute.  Third, perform 6 minutes of section C.  **Above 80% of HR_max_** | | | | | | 1. 500-meter row/cycling/run   10 air squats   1. 20 kettlebell swings   10 push-ups/pull-ups   1. 30 sit-ups   30 box step-ups | | | |  |
| **HIIT** | Make each bout as hard as possible. Return between bouts by walking calmly back to the starting point. Repeat 4-6 times.  **Above 90% of HR_max_** | | | | | | Choose one:   - Running/cycling/rowing - Stair-running - Uphill running | | | |  |
| **RESISTANCE TRAINING (once a week)** | | | | | | | | | | | |
| *Weeks 1-9* | | *Weeks 10-19* | | | *Weeks 20-26* | | | | *Repetitions (load)* | | |
| Leg press | | Leg press/back squat | | | Leg press/back squat | | | | 3 x 10, (75 % of 1RM) | | |
| Leg extension | | Bulgarian squats | | | Hip extensions | | | | 3 x 10, (75 % of 1RM) | | |
| Push-ups^a^ | | Cable seated row | | | Bent-over row | | | | 3 x 10, (75 % of 1RM) | | |
| Peck-deck | | Bench press | | | Bench press | | | | 3 x 10, (75 % of 1RM) | | |
| Lat pulldown | | Lat pulldown | | | Lat pulldown/Pull-ups | | | | 3 x 10, (75 % of 1RM) | | |
| Shoulder press | | Shoulder press | | | Shoulder press | | | | 3 x 10, (75 % of 1RM) | | |
| Abdominal crunches^a^ | | Abdominal crunches^a^ | | | Abdominal crunches^a^ | | | | 3 x 10 | | |

HR_max_: maximum heart rate.

1 RM: external load that can be lifted once i.e. one repetition maximum

^a^ Body weight exercise
