# Supplementary material for: Obesity is associated with increased brain glucose uptake and activity but not neuroinflammation (TSPO availability) in monozygotic twin pairs discordant for BMI—Exercise training reverses increased brain activity
Source: Diabetes Obes Metab. 2025 Sep 10;27(12):7097–109. doi: 10.1111/dom.70109 (PMC12587225; doi:10.1111/dom.70109)
Supplement: Supplementary file 2 — PET‐image analysis and modelling [file DOM-27-7097-s002.docx]

Supplementary file 2.

**PET-image analysis and modelling**

The obtained raw [^18^F]FDG-PET and [^11^C]PK11195-PET images were corrected for attenuation, dead time and decay. The block sequential regularized expectation maximization algorithm with BETA factor 150 for [^18^F]FDG and 350 for [^11^C]PK11195 were used for reconstruction.

For the radioligand data, PET images were processed using MAGIA pipeline^1^ in MATLAB (The Mathworks, Natick, MA), where PET data were first realigned frame-by-frame and co-registered to individual T1 weighted MRI images using SPM12 software (Wellcome Trust Centre for Neuroimaging, London, UK). [^18^F]FDG and [^11^C]PK11195-PET kinetic modelling was carried out for ROIs (whole brain, cortical grey matter, white matter, frontal cortex, parietal cortex, temporal cortex, occipital cortex, cingular cortex, posterior cingulate cortex, precuneus, hippocampus, putamen, thalamus and caudatus) that were parcellated using FreeSurfer software (version 6.0.0, <http://freesurfer.net/>). For [^18^F]FDG-PET data, BGU was quantified using arterial input Patlak method for 15–40 minute period.

For [^11^C]PK11195-PET data, regional TSPO availability was quantified as a distribution volume ratio (DVR), estimated with Logan’s method within 20–60 minutes, using pseudoreference region obtained using clustered reference algorithm^2^. In addition, parametric binding potential (BP_ND_) images were calculated using a basis function implementation of simplified reference tissue model with 250 basis functions. The resulting parametric maps were further normalized into MNI152 space in SPM12 and smoothed with Gaussian 8 mm FWHM filter.

**Resting state fMRI**

Resting state functional MRI data, to measure resting brain activity from BOLD signal, was acquired^3^. The scan was conducted twice and the participants had eyes open during the scan. A total of 197 functional volumes were acquired per scan and the mean of two scans was used for the analysis.

We used fMRIPrep 23.1.4 to preprocess the fMRI dataREF30. Anatomical T1 weighted reference images were processed with following steps: correction for intensity non-uniformity, skull-stripping, brain surface reconstruction, spatial normalization to the ICBM 152 Nonlinear Asymmetrical template version 2009c^4^ using nonlinear registration with antsRegistration (ANTs 2.2.0), and brain tissue segmentation. fMRI data were processed with following steps: co-registration to the T1 weighted reference image, slice-time correction, spatial smoothing with a 6-mm Gaussian kernel, automatic removal of motion artifacts using ICA-AROMA^5^, and resampling to the MNI152NLin2009cAsym standard space. Quality of images was assessed via the visual reports of fMRIPrep and was inspected manually in accord to the whole-brain field of view coverage, proper alignment to the anatomical images, and signal artifacts. All functional data were retained in the analysis.

**REFERENCES**

1. Karjalainen T, Tuisku J, Santavirta S, et al. Magia: Robust Automated Image Processing and Kinetic Modeling Toolbox for PET Neuroinformatics. *Front Neuroinform*. 2020;14:3. doi:10.3389/fninf.2020.00003

2. Schubert J, Tonietto M, Turkheimer F, Zanotti-Fregonara P, Veronese M. Supervised clustering for TSPO PET imaging. *Eur J Nucl Med Mol Imaging*. 2021;49(1):257-268. doi:10.1007/s00259-021-05309-z

3. Heiskanen MA, Honkala SM, Hentilä J, et al. Systemic cross-talk between brain, gut, and peripheral tissues in glucose homeostasis: effects of exercise training (CROSSYS). Exercise training intervention in monozygotic twins discordant for body weight. *BMC Sport Sci Med Rehabil*. 2021;13(1):16. doi:10.1186/s13102-021-00241-z

4. Fonov VS, Evans AC, McKinstry RC, Almli CR, Collins DL. Unbiased nonlinear average age-appropriate brain templates from birth to adulthood. *NeuroImage, suppl Suppl 1*. 2009;47. doi:https://doi.org/10.1016/S1053-8119(09)70884-5

5. Pruim RHR, Mennes M, van Rooij D, Llera A, Buitelaar JK, Beckmann CF. ICA-AROMA: A robust ICA-based strategy for removing motion artifacts from fMRI data. *Neuroimage*. 2015;112:267-277. doi:10.1016/j.neuroimage.2015.02.064
