# Supplementary material for: Obesity is associated with increased brain glucose uptake and activity but not neuroinflammation (TSPO availability) in monozygotic twin pairs discordant for BMI—Exercise training reverses increased brain activity
Source: Diabetes Obes Metab. 2025 Sep 10;27(12):7097–109. doi: 10.1111/dom.70109 (PMC12587225; doi:10.1111/dom.70109)
Supplement: Supplementary file 3 — Cognitive function test results before (PRE) and after (POST) the exercise intervention. [file DOM-27-7097-s004.docx]

Supplementary file 3. Cognitive function test results before (PRE) and after (POST) the exercise intervention. Data are expressed as model based mean [95 % CIs].

|  | Leaner co-twins | | Heavier co-twins | | P-value | | |
| --- | --- | --- | --- | --- | --- | --- | --- |
|  | **Pre** | **Post** | **Pre** | **Post** | **Baseline** | **Time** | **Time*group** |
|  | n = 11 | n = 10 | n = 12 | n = 11 |  |  |  |
| CERAD 10 Word list memory task type test |  |  |  |  |  |  |  |
| Round 1 (correct answers) | 6.34 [4.74; 7.95] | 6.54 [5.18; 7.90] | 6.00 [4.93; 7.07] | 6.24 [5.18; 7.31] | 0.682 | 0.676 | 0.954 |
| Round 2 (correct answers) | 7.14 [6.13; 8.15] | 7.69 [6.59; 8.80] | 7.08 [6.34; 7.83] | 8.12 [7.23; 9.02] | 0.765 | 0.096 | 0.402 |
| Round 3 (correct answers) | 7.82 [6.96; 8.68] | 8.32 [7.63; 9.02] | 8.25 [7.41; 9.09] | 8.94 [8.24; 9.63] | 0.445 | **0.035** | 0.721 |
| Vigilance test |  |  |  |  |  |  |  |
| Correct reactions (%) | 56.99 [45.21; 68.76] | 50.98 [39.39; 62.57] | 52.92 [39.63; 66.21] | 45.59 [32.98; 58.19] | 0.630 | 0.151 | 0.884 |
| Reaction time (ms) | 420.87 [413.10; 428.65] | 425.00 [418.12; 431.88] | 418.92 [405.22; 432.61] | 417.33 [406.30; 428.36] | 0.812 | 0.825 | 0.434 |
| Incorrect reactions | 3.62 [2.12;5.11] | 3.11 [1.21; 5.01] | 3.75 [1.81; 5.69] | 3.41 [0.98; 5.84] | 0.887 | 0.568 | 0.869 |
| N-back task (N=1) |  |  |  |  |  |  |  |
| Correct reactions (%) | 93.74 [89.72; 97.76] | 97.49 [91.76; 103.21] | 92.07 [76.69; 107.46] | 89.52 [74.16; 104.88] | 0.565 | 0.910 | 0.473 |
| Reaction time (ms) | 548.63 [471.15; 626.11] | 484.60 [400.48; 568.73] | 522.58 [438.09; 607.08] | 499.85 [415.84; 583.85] | 0.449 | 0.157 | 0.170 |
| Incorrect reactions | 6.38 [2.28; 10.48] | 2.56 [-3.28; 8.40] | 8.08 [-7.59; 23.76] | 10.69 [-4.97; 26.35] | 0.566 | 0.910 | 0.472 |
| N-back task (N=2) |  |  |  |  |  |  |  |
| Correct reactions (%) | 89.48 [85.95;93.02] | 89.94 [87.56; 92.31] | 85.07 [79.81; 90.32] | 88.48 [84.24; 92.72] | 0.176 | 0.234 | 0.281 |
| Reaction time (ms) | 655.70[556.66; 754.74] | 604.75 [512.66; 696.84] | 621.67 [551.70; 691.64] | 557.24 [487.98; 626.50] | 0.376 | 0.118 | 0.787 |
| Incorrect reactions | 10.73 [7.13; 14.34] | 10.27 [7.85; 12.69] | 15.33 [9.97; 20.70] | 11.74 [7.40; 16.09] | 0.166 | 0.223 | 0.264 |
| Reaction time test |  |  |  |  |  |  |  |
| Correct reactions (%) | 98.68 [97.44; 99.91] | 96.34 [94.42; 98.27] | 97.51 [95.13; 99.89] | 96.58 [92.89; 100.27] | 0.254 | 0.151 | 0.464 |
| Reaction time (ms) | 374.10 [344.54; 403.67] | 371.18 [327.09;415.27] | 360.58 [337.27; 383.90] | 364.83 [330.33;399.34] | 0.219 | 0.968 | 0.723 |
| Emotional sensitivity test |  |  |  |  |  |  |  |
| Unpleasant pictures  Unpleasant (0) – pleasant (100) | 14.11 [8.81; 19.40] | 16.95 [10.18; 23.72] | 11.11 [6.60; 15.61] | 13.33 [7.58; 19.07] | 0.241 | 0.100 | 0.782 |
| Unpleasant pictures  Peaceful (0) – agitated (100) | 68.40 [60.43; 76.37] | 68.08 [58.80; 77.37] | 76.16 [68.63; 83.69] | 71.81 [62.86; 80.77] | 0.110 | 0.270 | 0.235 |
| Neutral pictures  Unpleasant (0) – pleasant (100) | 55.79 [49.53; 62.05] | 57.64 [53.50; 61.78] | 57.62 [50.08; 65.16] | 57.80 [52.66; 62.94] | 0.611 | 0.580 | 0.408 |
| Neutral pictures  Peaceful (0) – agitated (100) | 41.65 [37.77;45.53] | 38.69 [33.45;43.92] | 39.38 [34.74; 44.01] | 35.42 [29.20; 41.65] | 0.322 | 0.115 | 0.748 |
| Pleasant pictures  Unpleasant (0) – pleasant (100) | 76.41 [71.78; 81.05] | 75.23 [68.54; 81.92] | 75.65 [71.12;80.18] | 71.72 [65.35; 78.09] | 0.649 | 0.446 | 0.331 |
| Pleasant pictures  Peaceful (0) – agitated (100) | 41.87 [35.80; 47.94] | 41.25 [33.65; 48.86] | 41.43 [37.29; 45.56] | 45.96 [40.59; 51.32] | 0.856 | 0.366 | **0.038** |
| Digit span text entry test |  |  |  |  |  |  |  |
| Longest digitspan | 7.21 [6.58; 7.84] | 7.05 [6.43; 7.67] | 7.08 [6.53; 7.63] | 7.20 [6.65; 7.75] | 0.504 | 0.937 | 0.499 |
| Fluid intelligence test |  |  |  |  |  |  |  |
| Correct reactions (easy) | 8.75 [7.90; 9.61] | 8.77 [7.76; 9.77] | 8.58 [7.91; 9.25] | 8.81 [8.06; 9.57] | 0.620 | 0.806 | 0.708 |
| Correct reactions (medium) | 24.10 [22.05; 26.15] | 27.82 [25.32; 30.32] | 24.50 [22.96; 26.04] | 26.91 [25.06; 28.77] | 0.721 | **0.009** | 0.436 |
| Correct reactions (hard) | 15.67 [13.53; 17.80] | 17.56 [15.58; 19.55] | 16.92 [15.06; 18.77] | 19.79 [18.16; 21.41] | 0.365 | **0.009** | 0.475 |
| Word retrieval test |  |  |  |  |  |  |  |
| Correct words | 7.09 [5.77; 8.42] | 7.88 [6.73; 9.02] | 7.08 [6.07; 8.10] | 8.28 [7.41; 9.16] | 0.872 | **0.001** | 0.330 |
| Word recognition |  |  |  |  |  |  |  |
| Correct answers | 18.84 [17.99; 19.70] | 19.48 [18.99;19.96] | 19.17 [18.56;19.77] | 19.83 [19.44;20.22] | 0.390 | **0.031** | 0.940 |

P-value for baseline describes the difference between heavier and leaner co-twins before exercise intervention. P-value for time describes the change from PRE to POST in all participants. P-value for time*group interaction describes the change difference between heavier and leaner co-twins from pre to post. **CERAD 10 Word list memory task type test**: Ten words were shown on computer screen to participants one at a time. When all the words were shown, participants wrote the words they remembered were shown. This was repeated for three rounds and the correct answers are reported for each round. **Vigilance test:** Different letters were shown on the computer screen on at a time. Whenever a letter X was shown, participant pressed “Space” as quick as possible. Correct reactions, reaction time and incorrect reactions are reported. **N-back (N=1, N=2) tasks.** Different letters were shown on the computer screen on at a time. If the letter was same as the previous one (N=1 or N=2), F button is pressed and if it is not the same, J is pressed. **Reaction time test**: Pictures of thumbs appeared one at a time to the left or right side of the screen into a box. If the thumb appeared on the left side of the screen, F-button was pressed and if it appeared on the right side J-button was pressed. **Emotional sensitivity test.** Unpleasant, neutral and pleasant pictures were shown to participants. Participants answered how pleasant (Scale: Unpleasant = 0; Pleasant = 100) and agitated (Scale: Peaceful = 0; Agitated = 100) they felt when looking at the pictures. **Digit span entry test.** A string of digits was shown to participants one digit at a time. After the complete string of digits was shown, participants wrote the string of digits in correct order. **Fluid intelligence test.** A matrix of 3 x 3 with different shapes was shown to participants. One shape from the 3 x 3 matrix was missing. Participant had to choose from four options which shape fit the matrix best. This task had three degrees of difficulty (easy, medium and hard). **Word retrieval test.** Participants recalled as many words as possible from the CERAD 10 Word list memory task type test after completing vigilance test, N-back (N=1, N=2) tasks**,** reaction time test, emotional sensitivity test, digit span entry test and fluid intelligence test. **Word recognition.** Words were shown on the computer screen one at a time and participant answered whether the word shown was part of the word list that war learned in CERAD 10 Word list memory task type test. The tasks were done at one time in a following order: CERAD 10 Word list memory task type test, vigilance test, N-back (N=1, N=2) tasks, reaction time test, emotional sensitivity test, digit span entry test, fluid intelligence test, word retrieval test and word recognition test.
