# Supplementary material for: Obesity is associated with increased brain glucose uptake and activity but not neuroinflammation (TSPO availability) in monozygotic twin pairs discordant for BMI—Exercise training reverses increased brain activity
Source: Diabetes Obes Metab. 2025 Sep 10;27(12):7097–109. doi: 10.1111/dom.70109 (PMC12587225; doi:10.1111/dom.70109)
Supplement: Supplementary file 4 — Blood glucose and insulin levels measured during euglycaemic hyperinsulinemic clamp before (PRE) and after (POST) the exercise intervention. [file DOM-27-7097-s005.docx]

Supplementary file 4. Blood glucose and insulin levels measured during euglycaemic hyperinsulinemic clamp before (PRE) and after (POST) the exercise intervention. Data are expressed as model based mean [95 % CIs].

|  | Leaner co-twins | | Heavier co-twins | | p-value | | |
| --- | --- | --- | --- | --- | --- | --- | --- |
|  | **PRE (n=11)** | **POST (n=10)** | **PRE (n=11)** | **POST (n=9)** | **Baseline** | **Time** | **Time x Group** |
| Steady state mean blood glucose (mmol/l) | 5.05 [4.89;5.22] | 4.95 [4.78;5.12] | 4.90 [4.67;5.13] | 4.97 [4.69;5.25] | 0.265 | 0.861 | 0.418 |
| Blood glucose 0’ (mU/l) | 5.43 [5.12;5.74] | 5.60 [5.36;5.84] | 5.57 [5.21;5.93] | 5.77 [5.49;6.06] | 0.351 | 0.182 | 0.861 |
| Blood glucose 60’ (mU/l) | 4.70 [4.32;5.07] | 4.47 [3.91;5.03] | 4.75 [4.43;5.08] | 4.39 [3.89;4.89] | 0.817 | 0.187 | 0.735 |
| Blood glucose 120’ (mU/l) | 5.15 [4.90;5.39] | 5.03 [4.69;5.36] | 5.03 [4.74;5.31] | 5.21 [4.81;5.60] | 0.450 | 0.875 | 0.312 |
| Blood insulin 0’ (mU/l) | 7.7 [5.8;9.5] | 8.1 [4.3;12.0] | 10.6 [7.9;13.4] † | 14.0 [8.1;19.8] | **0.030** | 0.299 | 0.220 |
| Blood insulin 60’ (mU/l) | 63.5 [54.5;72.5] † | 67.9 [60.2;75.6] | 65.8 [57.2;74.4] ‡ | 70.4 [62.6;78.3] | 0.549 | 0.170 | 0.961 |
| Blood insulin 120’ (mU/l) | 68.2 [59.7;76.7] | 71.7 [65.1;78.3] | 65.9 [58.5;73.2] † | 76.8 [70.2;83.4] | 0.648 | **0.022** | 0.091 |

Time points 0’, 60’ and 120’ depict minutes from the start of the euglycaemic hyperinsulinemic clamp. P-value for baseline describes the difference between heavier and leaner co-twins before exercise intervention. P-value for time describes the change from PRE to POST in all participants. P-value for time*group interaction describes the change difference between heavier and leaner co-twins from pre to post. †=n=10, ‡=n=9.
