# Supplementary material for: Obesity is associated with increased brain glucose uptake and activity but not neuroinflammation (TSPO availability) in monozygotic twin pairs discordant for BMI—Exercise training reverses increased brain activity
Source: Diabetes Obes Metab. 2025 Sep 10;27(12):7097–109. doi: 10.1111/dom.70109 (PMC12587225; doi:10.1111/dom.70109)
Supplement: Supplementary file 5 — Insulin‐stimulated glucose uptake and TSPO availability in different regions of interest (ROI) of brain before (PRE) and after (POST) the intervention. [file DOM-27-7097-s001.docx]

Supplementary file 5. Insulin-stimulated glucose uptake in different regions of interest (ROI) of brain before (PRE) and after (POST) the intervention

| GU (µmol/100g/min) | Leaner co-twins | | Heavier co-twins | | P-value | | |
| --- | --- | --- | --- | --- | --- | --- | --- |
|  | **Pre (n=11)** | **Post (n=10)** | **Pre (n=8)** | **Post (n=8)** | **Baseline** | **Time** | **Time*group** |
| Whole brain | 22.6 [20.5; 24.6] | 22.1 [19.1; 25.2] | 24.4 [22.4; 26.5] | 24.1 [21.3; 26.9] | 0.111 | 0.670 | 0.889 |
| Cortical grey matter | 27.4 [24.9; 29.9] | 26.8 [23.0; 30.6] | 30.2 [27.6; 32.8] | 29.6 [26.0; 33.2] | 0.078 | 0.574 | 0.992 |
| White matter | 18.8 [17.1; 20.5] | 18.5 [15.9; 21.0] | 20.2 [18.5; 21.9] | 20.1 [17.7; 22.4] | 0.151 | 0.765 | 0.786 |
| Frontal Cortex | 28.5 [25.7; 31.2] | 27.7 [23.6; 31.8] | 31.4 [28.6; 34.2] | 30.9 [27.0; 34.7] | 0.081 | 0.544 | 0.873 |
| Parietal Cortex | 28.7 [26.2; 31.3] | 27.8 [23.8; 31.8] | 32.2 [29.6; 34.9] | 31.5 [27.6; 35.4] | **0.032** | 0.433 | 0.886 |
| Temporal Cortex | 24.7 [22.4; 26.9] | 24.4 [21.2; 27.6] | 26.8 [24.4; 29.2] | 26.2 [23.0; 29.4] | 0.128 | 0.642 | 0.809 |
| Occipital Cortex | 29.0 [26.1; 31.9] | 28.9 [24.7; 33.2] | 31.4 [28.6; 34.1] | 30.9 [27.2; 34.5] | 0.141 | 0.829 | 0.800 |
| Cingular Cortex | 27.6 [24.8; 30.4] | 27.3 [23.2; 31.4] | 30.1 [27.4; 32.7] | 29.5 [25.9; 33.1] | 0.155 | 0.653 | 0.899 |
| Hippocampus | 17.8 [16.4; 19.1] | 17.2 [15.4; 19.1] | 18.9 [17.3; 20.4] | 18.5 [16.4; 20.6] | 0.158 | 0.522 | 0.871 |
| Putamen | 30.9 [27.7; 34.2] | 30.3 [25.3; 35.4] | 33.8 [30.7; 36.9] | 33.8 [29.3; 38.2] | 0.163 | 0.794 | 0.765 |
| Thalamus | 24.3 [21.6; 27.0] | 24.2 [20.5; 27.8] | 26.3 [23.4; 29.3] | 26.1 [22.5; 29.8] | 0.193 | 0.854 | 0.961 |
| Caudatus | 27.3 [24.3; 30.3] | 26.3 [21.4; 31.3] | 30.2 [28.0; 32.5] | 29.7 [26.4; 33.1] | **0.043** | 0.435 | 0.756 |

GU = Insulin stimulated glucose uptake. Data are expressed as model based means [95 % CIs]

PK binding to TSPO in different regions of interest (ROI) in brain before (PRE) and after (POST) the intervention

| PK DVR | Leaner co-twins | | Heavier co-twins | | P-value | | |
| --- | --- | --- | --- | --- | --- | --- | --- |
|  | **Pre (n=10)** | **Post (n=9)** | **Pre (n=8)** | **Post (n=8)** | **Baseline** | **Time** | **Time*group** |
| Whole brain | 1.101 [1.085; 1.117] | 1.108 [1.091; 1.125] | 1.088 [1.066; 1.109] | 1.089 [1.068; 1.111] | 0.168 | 0.386 | 0.440 |
| Cortical grey matter | 1.114 [1.094; 1.134] | 1.121 [1.100; 1.142] | 1.113 [1.085; 1.141] | 1.113 [1.087; 1.140] | 0.898 | 0.567 | 0.380 |
| White matter | 1.077 [1.057; 1.097] | 1.080 [1.055; 1.105] | 1.051 [1.031; 1.071] | 1.059 [1.035; 1.084] | **0.031** | 0.329 | 0.410 |
| Frontal Cortex | 1.079 [1.057; 1.102] | 1.088 [1.064; 1.111] | 1.081[1.051; 1.111] | 1.082[1.052; 1.111] | 0.676 | 0.492 | 0.313 |
| Parietal Cortex | 1.119 [1.098; 1.140] | 1.120 [1.098; 1.142] | 1.125 [1.097; 1.152] | 1.123 [1.096; 1.149] | 0.576 | 0.928 | 0.700 |
| Temporal Cortex | 1.124 [1.101; 1.147] | 1.135 [1.115; 1.156] | 1.118 [1.073; 1.163] | 1.121 [1.086; 1.157] | 0.471 | 0.273 | 0.429 |
| Occipital Cortex | 1.203 [1.175; 1.232] | 1.205 [1.171; 1.239] | 1.201 [1.171; 1.231] | 1.202 [1.170; 1.234] | 0.804 | 0.900 | 0.897 |
| Cingular Cortex | 1.106 [1.075; 1.137] | 1.118 [1.088; 1.147] | 1.092 [1.057; 1.127] | 1.095 [1.065; 1.125] | 0.147 | 0.409 | 0.396 |
| Hippocampus | 1.089 [1.061; 1.116] | 1.112 [1.083; 1.141] | 1.054 [1.023; 1.085] | 1.057 [1.026; 1.089] | **0.032** | 0.128 | 0.162 |
| Putamen | 1.193 [1.164; 1.222] | 1.202[1.171; 1.233] | 1.167[1.126; 1.208] | 1.167 [1.125; 1.210] | 0.116 | 0.654 | 0.522 |
| Thalamus | 1.269 [1.232; 1.307] | 1.296 [1.245; 1.347] | 1.294 [1.250; 1.338] | 1.273 [1.219; 1.327] | 0.314 | 0.842 | 0.058 |
| Caudatus | 0.926 [0.900; 0.952] | 0.924 [0.892; 0.955] | 0.933[0.894; 0.972] | 0.928 [0.889; 0.968] | 0.609 | 0.612 | 0.834 |

Data are expressed as model based means [95 CIs].
