# Supplementary material for: Obesity is associated with increased brain glucose uptake and activity but not neuroinflammation (TSPO availability) in monozygotic twin pairs discordant for BMI—Exercise training reverses increased brain activity
Source: Diabetes Obes Metab. 2025 Sep 10;27(12):7097–109. doi: 10.1111/dom.70109 (PMC12587225; doi:10.1111/dom.70109)
Supplement: Supplementary file 6 — Statistically significant Pearson correlation coefficients between translocator protein (TSPO) availability measured by PK distribution volume ratio (PK DVR) and main outcome measures at baseline (Pre). [file DOM-27-7097-s003.docx]

**Supplementary file 6. Statistically significant Pearson correlation coefficients between translocator protein (TSPO) availability measured by PK distribution volume ratio (PK DVR) and main outcome measures at baseline (Pre)**

|  | PK DVR Hippocampus | PK DVR White matter | PK DVR Whole brain |
| --- | --- | --- | --- |
| M-value | r=0.66, p=0.003 | r=0.48, p=0.042 | r=0.44, p=0.067 |
| VO_2peak_ | r=0.53, p=0.024 | r=0.69, p=0.002 | r=0.61, p=0.008 |
| hs-CRP | r=-0.72, p=0.001 | r=-0.66, p=0.004 | r=-0.71, p=0.001 |
| BMI | r=-0.74, p<0.001 | r=-0.71, p=0.001 | r=-0.74, p=0.002 |
| Visceral fat mass | r=-0.73, p<0.001 | r=-0.82, p<0.001 | r=-0.52, p=0.033 |

Abbreviations: M-value=whole-body insulin sensitivity, VO_2peak_=cardiorespiratory fitness, hs-CRP=high sensitive C-reactive protein
